# Assessment of broth disk elution method for aztreonam in combination with ceftazidime/avibactam against *Enterobacterales* isolates

**DOI:** 10.1128/spectrum.00953-24

**Published:** 2024-09-03

**Authors:** Peipei Song, Jian Xu, Lan Jiang, Qin Zhang, Chenggui Liu

**Affiliations:** 1 Department of Laboratory, Chengdu Womenʼs and Childrenʼs Central Hospital, School of Medicine, University of Electronic Science and Technology of China, Chengdu, China; 2 Department of Laboratory, Urumqi Maternal and Child Health Care Hospital, Urumqi, China; 3 Department of Public Health, Chengdu Womenʼs and Childrenʼs Central Hospital, School of Medicine, University of Electronic Science and Technology of China, Chengdu, China; Banner Health Pathology, Tucson, Arizona, USA

**Keywords:** *Enterobacterales*, aztreonam, ceftazidime/avibactam, antimicrobial susceptibility testing, broth disk elution method

## Abstract

The combination of aztreonam with ceftazidime/avibactam is considered a potential therapeutic approach for the treatment of infections caused by metallo-β-lactamase (MBL)-producing isolates. In this study, *in vitro* antibacterial activity of aztreonam with avibactam against 204 carbapenemase-producing *Enterobacterales* was determined by broth disk elution (BDE) method of two detection volumes (5- and 2-mL broth), with broth microdilution (BMD) method as a reference. For the BDE-5mL test, the categorical agreement (CA) of ATM+CZA_-lo_ tube (aztreonam/ceftazidime/avibactam: 6/6/4 mg/L) was 99.5%, with 0.5% major error (ME) and 0% very major error (VME); the CA of 2ATM+CZA_-lo_ tube (12/6/4 mg/L) was 100%, with no ME and VME. For the BDE-2mL test, the CA of ATM+2CZA_-hi_ tube (15/10/4 mg/L) was 98.5%, with 0% ME and 37.5% VME; the CA of 2ATM+2CZA_-hi_ tube (30/10/4 mg/L) was 97.1%, with 0% ME and 75% VME. The BDE-5 mL test is an economical and practical method for clinical microbiology laboratories to determine the antibacterial susceptibility of aztreonam with avibactam against *Enterobacterales*, especially the 2ATM+CZA_-lo_ tube with a final concentration of 12/6/4 mg/L of aztreonam/ceftazidime/avibactam.

**IMPORTANCE:**

Infections caused by metallo-β-lactamase (MBL)-producing *Enterobacterales* are increasingly reported worldwide, and it is a significant challenge for clinical infection treatment. MBLs are adept at hydrolyzing almost all traditional β-lactam antibiotics except aztreonam, and the enzyme activity cannot be inhibited by traditional or novel β-lactamase inhibitors. The good thing is that the combination of aztreonam with ceftazidime/avibactam has been proven to be one of the potential therapeutic approaches for treating infections related with MBL-producing isolates. Broth microdilution (BMD) method is recommended as a reference method for its accuracy, but it is too complex to perform in most routine laboratories. Finding a more convenient, practical, and accurate susceptibility testing method for aztreonam/avibactam in clinical microbiology laboratories is very necessary. Here, we evaluated the performance of broth disk elution (BDE) method for aztreonam in combination with ceftazidime/avibactam against *Enterobacterales* isolates, with BMD as a reference.

## INTRODUCTION

Carbapenem-resistant *Enterobacterales* (CRE) have rapidly spread worldwide, and the infections caused by these pathogenic bacteria have posed significant challenges for clinical treatment (1). Production of carbapenemase is the most important resistance mechanism to carbapenems in *Enterobacterales* (2). As we have known, in addition to *Klebsiella pneumoniae* carbapenemase (KPC), metallo-β-lactamases (MBLs) are another category of the most popular carbapenemases in the world, mainly including the New Delhi MBL (NDM), followed by imipenemases (IMP) and Verona integron-encoded MBL (VIM) (3–5). All MBLs are able to hydrolyze almost all traditional β-lactam antibiotics except aztreonam, as well as the traditional or novel β-lactamase inhibitors such as clavulanic acid, sulbactam, tazobactam, avibactam, vabobactam, and relebactam. Due to the very limited available antimicrobial therapy, there was a high mortality for the patients infected by MBL-producing strains (6), as well as a heavy burden of treatment costs. Although aztreonam cannot be hydrolyzed by MBLs, the MBL-producing strains usually co-express one or more other β-lactamases, which can hydrolyze aztreonam, such as extended spectrum β-lactamases (ESBLs) or AmpC, making the MBL-producing strains still resistant to aztreonam (7). The good thing is that the combination of aztreonam with avibactam has been proven to be one of the potential therapeutic approaches for treating infections related with MBL-producing isolates (8–10). However, aztreonam/avibactam is not available commercially. Previous studies have shown that ceftazidime did not affect the antibacterial activity of aztreonam/avibactam against strains in the combination of aztreonam with ceftazidime/avibactam, so the antibacterial activity of aztreonam/avibactam could be achieved through the combination of aztreonam with ceftazidime/avibactam (11). Mikhail et al. and Hobson et al. showed that the combination of aztreonam with ceftazidime/avibactam has synergistic antibacterial activity against multidrug-resistant *K. pneumoniae* and demonstrates good therapeutic efficacy in treating bloodstream infections caused by NDM-1-producing *Morganella morganii* (12, 13). Falcone et al. also showed that the combination of aztreonam with ceftazidime/avibactam could reduce 30-day mortality in patients with bloodstream infections caused by MBL-producing *Enterobacterales*, significantly shorten the length of hospital stay, and reduce the risk of clinical treatment failure within 14 days (14). In addition, the Infectious Diseases Society of America has also recommended the combination of aztreonam with ceftazidime/avibactam as an alternative treatment for MBL-producing CRE infections with limited therapeutic options (15).

Therefore, it is urgent to clarify the susceptibility of bacteria to aztreonam/avibactam for a more rational application in the treatment of infections related to MBL-producing strains. Broth microdilution (BMD) method is recommended as a reference method by the Clinical and Laboratory Standards Institute (CLSI) for its accuracy, but it is too complex to perform in most routine laboratories. More recently, a MIC strip has been developed by Liofilchem to directly determine the MIC of aztreonam/avibactam (16), but it is not available in most countries, especially in China. Finding a more convenient, practical, and accurate susceptibility testing method for aztreonam/avibactam in clinical microbiology laboratories is very necessary. Broth disk elution (BDE) method is another acceptable method endorsed by the CLSI in 2024 for determining the susceptibility of *Enterobacterales* to aztreonam plus ceftazidime/avibactam based on the study of Harris et al., which combines the advantages of both disk diffusion and broth microdilution method, however, Harris et al. only utilized 5-mL tubes of cation-adjusted Mueller Hinton broth (CAMHB) with 30-μg aztreonam disks and 30/20-μg ceftazidime-avibactam disks to evaluate the performance of BDE for aztreonam in combination with ceftazidime/avibactam against *Enterobacterales* and *Stenotrophomonas maltophilia*, and the final concentration of aztreonam/ceftazidime/avibactam evaluated was 6/6/4 mg/L (17, 18). In this study, we utilized the BDE method in 5-mL CAMHB at final concentrations of 6/6/4 mg/L and 12/6/4 mg/L aztreonam/ceftazidime/avibactam, respectively, as well as the BDE method with 2-mL CAMHB at final concentrations of 15/10/4 mg/L and 30/10/4 mg/L aztreonam/ceftazidime/avibactam, respectively, spanning approximately two log2-dilution range of expected aztreonam/avibactam pharmacokinetics/pharmacodynamics (PK/PD) breakpoints to fully evaluate the BDE performance, filling in some of the existing gaps left after the studies of Harris et al. and Khan et al. (18, 19).

## MATERIALS AND METHODS

### Clinical isolates

A total of 204 non-duplicated *Enterobacterales* were collected from 16 hospitals in 31 provinces or municipalities across China in 2021. Species were identified using matrix-assisted laser desorption ionization-time of flight mass spectrometry (MALDI-TOF MS) system (bioMérieux, France). All *Enterobacterales* isolates were carbapenem-resistant isolates, and the carbapenemase genes were detected by whole-genome sequencing (55 harboring *bla*_NDM-1_, 54 harboring *bla*_NDM-5_, 28 harboring *bla*_KPC-2_, 24 co-harboring *bla*_NDM_ and *bla*_KPC_, 7 harboring *bla*_IMP-4_, 6 co-harboring *bla*_NDM_ and *bla*_IMP_, 5 harboring *bla*_NDM-9_, 4 harboring *bla*_KPC-33_, 4 co-harboring *bla*_IMP_ and *bla*_KPC_, 4 harboring *bla*_IMP-6_, 3 harboring *bla*_IMP-8_, 2 harboring *bla*_KPC-71_, 2 harboring *bla*_NDM-13_, 2 harboring *bla*_VIM-1_, 2 co-harboring *bla*_NDM_, *bla*_KPC_, and *bla*_IMP_ , 1 harboring *bla*_KPC-135_, and 1 harboring *bla*_VIM-4_) (Table 1).

**TABLE 1:** Detection of carbapenemase genes among 204 *Enterobacterales*

Species	Carbapenemase gene (no. of isolates)
*Klebsiella pneumoniae* (*n* = 80)	*bla*_KPC-2_ (27), *bla*_KPC+NDM_ (19), *bla*_NDM-1_ (10), *bla*_NDM-5_ (9), *bla*_KPC-33_ (4), *bla*_IMP-4_ (3), *bla*_KPC+IMP_ (2), *bla*_KPC-71_ (2), *bla*_NDM-9_ (2), *bla*_IMP-8_ (1), *bla*_KPC-135_ (1)
*Escherichia coli* (*n* = 46)	*bla*_NDM-5_ (34), *bla*_NDM-1_ (7), *bla*_NDM-9_ (3), *bla*_NDM-13_ (2)
*Enterobacter cloacae* (*n* = 35)	*bla*_NDM-1_ (11), *bla*_NDM-5_ (7), *bla*_NDM+IMP_ (5), *bla*_IMP-26_ (4), *bla*_IMP-8_ (2), *bla*_KPC+IMP_ (2), *bla*_VIM-1_ (2), *bla*_VIM-4_ (1), *bla*_IMP-4_ (1)
*Klebsiella oxytoca* (*n* = 16)	*bla*_NDM-1_ (7), *bla*_KPC+NDM_ (3), *bla*_KPC+NDM+IMP_ (2), *bla*_IMP-4_ (2), *bla*_NDM+IMP_ (1), *bla*_NDM-5_ (1)
*Citrobacter freundii* (*n* = 11)	*bla*_NDM-1_ (7), *bla*_KPC+NDM_ (2), *bla*_NDM-5_ (2)
*Klebsiella aerogenes* (*n* = 7)	*bla*_NDM-1_ (6), *bla*_NDM-5_ (1)
*Citrobacter koseri* (*n* = 2)	*bla*_NDM-1_ (2)
*Citrobacter portucalensis* (*n* = 2)	*bla*_NDM-1_ (2)
*Raoultella planticola* (*n* = 2)	*bla*_IMP-4_ (1), *bla*_NDM-1_ (1)
*Providencia rettgeri* (*n* = 1)	*bla*_KPC-2_ (1)
*Raoultella ornithinolytica* (*n* = 1)	*bla*_NDM-1_ (1)
*Serratia marcescens* (*n* = 1)	*bla*_NDM-1_ (1)

### Culture medium and reagents

Aztreonam (lot: 130507-201303) and avibactam (lot: 130909) for broth microdilution were from the China Institute for Food and Drug Control (CFDC) and MedChemExpress (MCE) Biotechnology Inc., respectively. The disks of ceftazidime/avibactam (30/20 µg, lot: 508389 and 10/4 µg, lot: 488996) and aztreonam (30 µg, lot: 3495785) were from the Mast Group Ltd. (UK) and Oxoid Ltd. (UK), respectively. The CAMHB (lot: 1079890) was from the Becton, Dickinson and Company (USA).

### Broth microdilution method

All *Enterobacterales* isolates were tested for susceptibility to aztreonam, ceftazidime/avibactam, and aztreonam/avibactam by BMD method according to CLSI M07 of 2018 (20). The concentration range of all tested drugs was from ≤0.06 to >128 mg/L. According to the CLSI breakpoints for *Enterobacterales*, a MIC of ≤4 mg/L was susceptible to aztreonam, whereas a MIC of ≥16 mg/L was resistant; a MIC of ≤8/4 mg/L was considered susceptible to ceftazidime/avibactam, whereas a MIC of ≥16/4 mg/L was resistant (17). MICs of ≤8/4 mg/L and ≥16/4 mg/L were considered susceptible and resistant to aztreonam/avibactam (avibactam for a constant concentration of 4 mg/L) according to the PK/PD breakpoints for *Enterobacterales*, respectively (10, 21–23). The *Escherichia coli* ATCC 25922 and *K. pneumoniae* ATCC 700603 strains were used for quality control (QC) on each day of testing. The expected MIC QC ranges of *E. coli* ATCC 25922 for aztreonam, ceftazidime/avibactam, and aztreonam/avibactam were 0.06–0.5 mg/L, 0.06/4–0.5/4 mg/L, and 0.03/4–0.12/4 mg/L, respectively; and the expected MIC QC ranges of *K. pneumoniae* ATCC 700603 for aztreonam, ceftazidime/avibactam, and aztreonam/avibactam were >8 mg/L, 0.25/4–2/4 mg/L, and 0.06/4–0.5/4 mg/L, respectively (17).

### Broth disk elution method

The BDE test was performed in parallel with the reference BMD method. Different amounts of aztreonam disks and ceftazidime/avibactam disks were placed into the broth and were dissolved thoroughly to simulate the gradient concentration of broth dilution.

### Culture medium preparation

**Fig 1:**
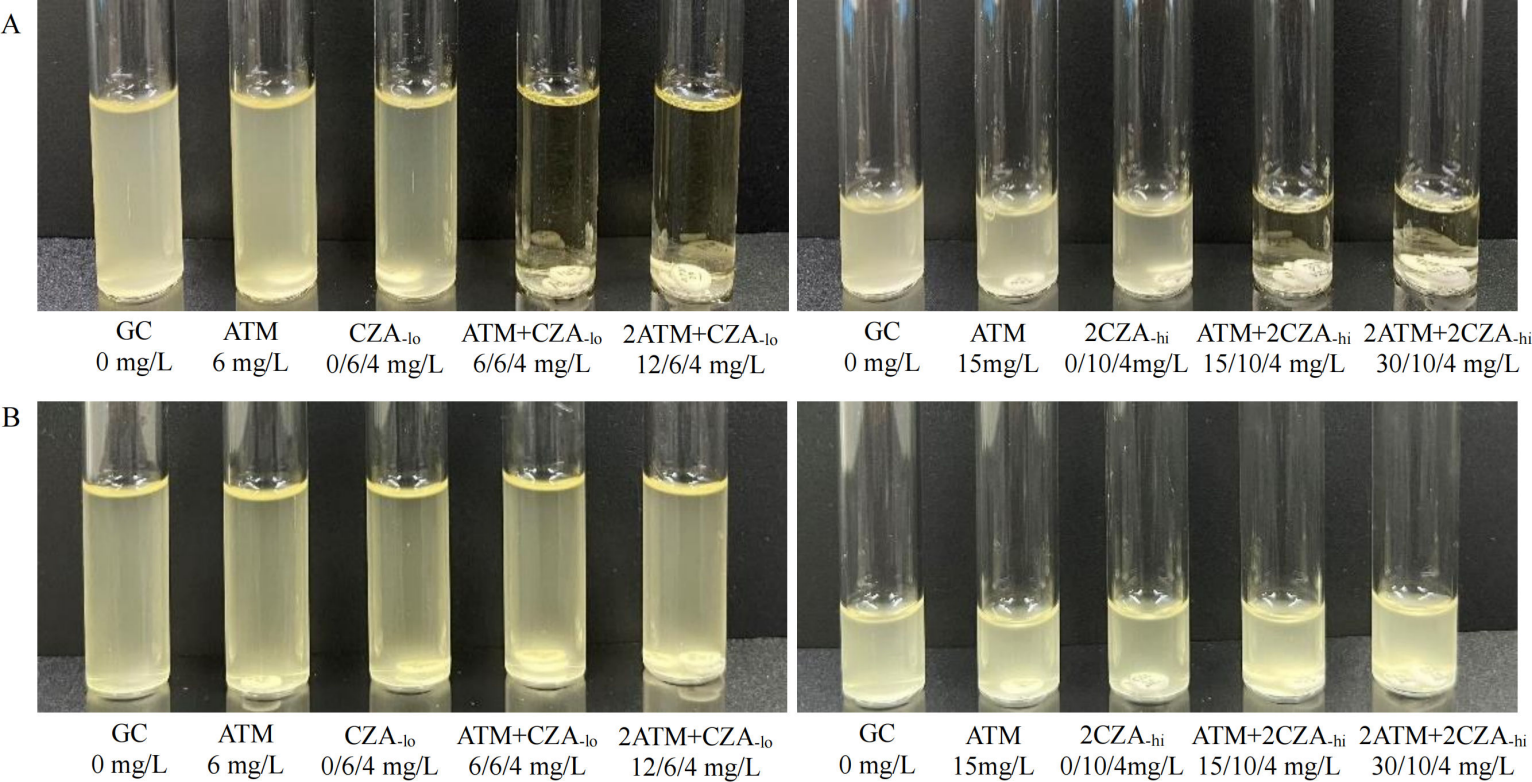
Susceptibility testing results by BDE for CZA in combination with ATM. (A) The phenotypic outcome for an *E. coli* (strain number: A17) by BDE of two detection volumes (left: 5 mL, right: 2 mL), which was resistant (growth) to both of the ATM tubes (6 and 15 mg/L) and CZA tubes (0/6/4 mg/L and 0/10/4 mg/L) but susceptible (no growth) to the ATM-CZA combination tubes (6/6/4 mg/L, 12/6/4 mg/L, 15/10/4 mg/L, and 30/10/4 mg/L). (B) The phenotypic outcome for a *Providencia rettgeri* strain (strain number: A38) by BDE of two detection volumes (left: 5 mL, right: 2 mL), which was resistant (growth) to each of the ATM tubes (6 and 15 mg/L), CZA tubes (0/6/4 mg/L and 0/10/4 mg/L), or ATM-CZA combination tubes (6/6/4 mg/L, 12/6/4 mg/L, 15/10/4 mg/L, and 30/10/4 mg/L). GC, growth control; ATM, aztreonam; CZA, ceftazidime/avibactam.

For BDE with 5-mL broth (BDE-5mL): 5-mL CAMHB was added to each of five separate sterile tubes. Aztreonam disk (30 µg) and ceftazidime/avibactam disk (30/20 µg) were added to each sterile tube with the following marks: no disk (marked as the growth control [GC] tube), one 30-µg aztreonam disk (marked as ATM tube), one 30/20-µg ceftazidime/avibactam disk (marked as CZA_-lo_ tube), one 30-µg aztreonam disk and one 30/20-µg ceftazidime/avibactam disk (marked as ATM+CZA_-lo_ tube), two 30-µg aztreonam disks and one 30/20-µg ceftazidime/avibactam disk (marked as 2ATM+CZA_-lo_ tube), respectively. The final concentrations of aztreonam/ceftazidime/avibactam in each tube were 0/0/0 mg/L, 6/0/0 mg/L, 0/6/4 mg/L, 6/6/4 mg/L, and 12/6/4 mg/L, respectively (Fig. 1, left). For BDE with 2-mL broth (BDE-2mL): 2-mL CAMHB was added to each of five separate sterile tubes. Aztreonam disk (30 µg) and ceftazidime/avibactam disk (10/4 µg) were added to each of five sterile culture tubes with the following marks: no disk (marked as GC tube), one 30-µg aztreonam disk (marked as ATM tube), two 10/4-µg ceftazidime/avibactam disks (marked as 2CZA_-hi_ tube), one 30-µg aztreonam disk and two 10/4-µg ceftazidime/avibactam disks (marked as ATM+2CZA_-hi_ tube), two 30-µg aztreonam disks and two 10/4-µg ceftazidime/avibactam disks (marked as 2ATM+2CZA_-hi_ tube), respectively. The final concentrations of aztreonam/ceftazidime/avibactam in each tube were 0/0/0 mg/L, 15/0/0 mg/L, 0/10/4 mg/L, 15/10/4 mg/L, and 30/10/4 mg/L, respectively (Fig. 1, right).

### Inoculum preparation

All tubes were incubated at room temperature for 30–60 minutes to ensure the medicine on the disks fully elute into the CAMHB. The same 0.5 McFarland standard inoculum as used for the BMD was prepared by suspending three to five fresh colonies from an overnight blood agar plate in 3-mL saline tubes. Twenty-five or 10µL 0.5 McFarland bacterial suspension was added to 5-mL CAMHB tubes and 2-mL CAMHB tubes, respectively (the final inoculum content was around 7.5 × 10^5^ CFU/mL).

### Results interpretation

All tubes were incubated at 35°C for 16–20 hours under ambient air, and were then read to determine whether there was a visible turbidity in each tube. Except for the GC tubes, if the broth was clear (no growth in the tube), the strain was susceptible to the tested antibiotics; otherwise, it was resistant. The quality control strains were *E. coli* ATCC 25922, *K. pneumoniae* ATCC BAA-1705, and *K. pneumoniae* ATCC BAA-2146.

### Statistical analysis

According to the breakpoints, the susceptibility and resistance of *Enterobacterales* to aztreonam/avibactam were calculated for each method. Compared to the reference method, categorical agreement (CA) rates of ≥90%, with less than 3% for major errors (MEs) and 1.5% for very major errors (VMEs), are considered acceptable according to CLSI M52 (24). When a categorical error (such as VME or ME) was observed, a repeat testing should be conducted at least once by repeating the BMD and the BDE in parallel, with a newly prepared inoculum for both tests. When the repeat testing resolved the error, the repeat testing result should be considered as the final result. If on the repeat testing the result confirmed the initial result (the repeat MIC values were within ±1 log2 dilution but a categorical error remained), the error was deemed to be a confirmed categorical error (25), and only the results confirmed by the repeat testing should be included in the final statistical analysis.

## RESULTS

**Fig 2:**
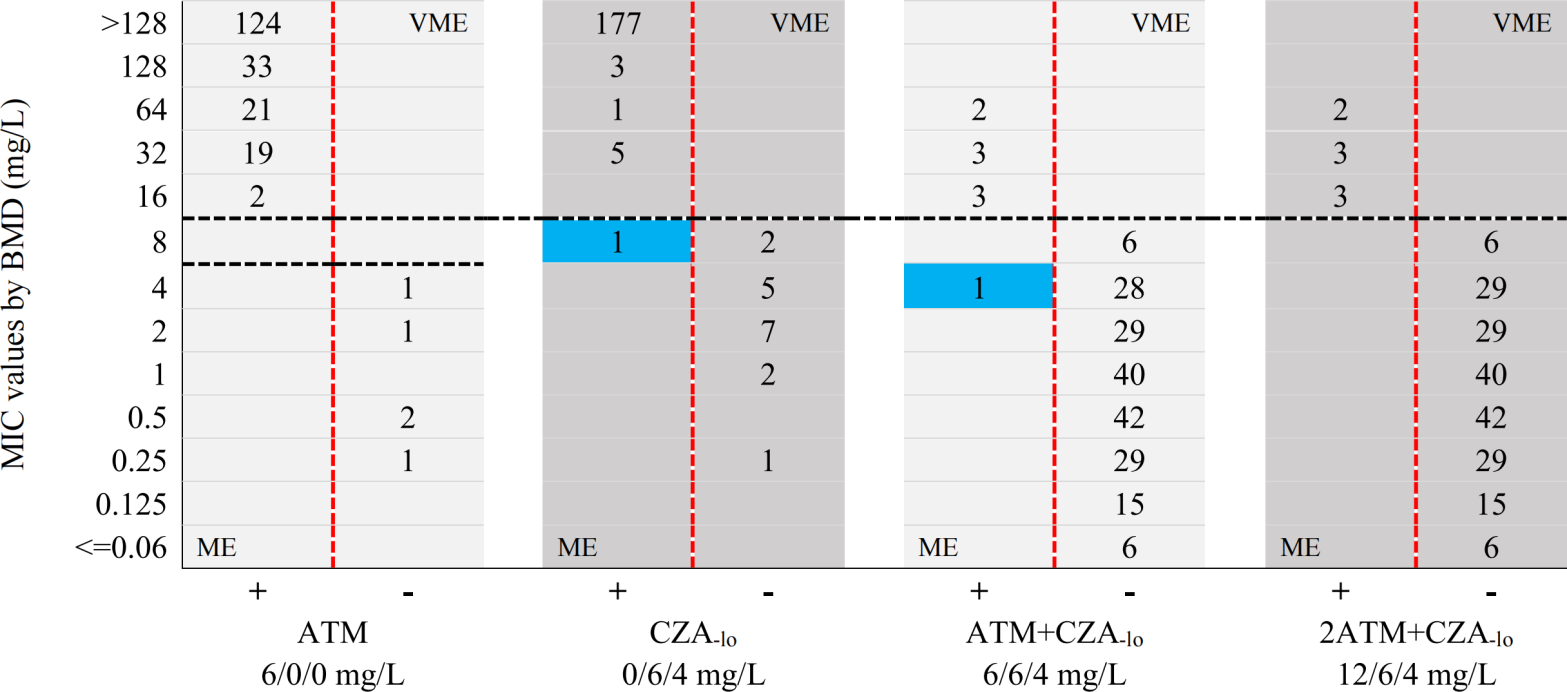
Comparsion scattergram between MIC values by BMD and phenotypic outcome by BDE-5mL against 204 *Enterobacterales* isolates. Black dash lines indicate the CLSI breakpoints for ATM and CZA, and the PK/PD breakpoints for ATM/AVI. Red dash lines are the boundary of growth (+) and no-growth (–) phenotypic outcomes by BDE. Blue backgrounds indicate the MEs observed in BDE. ATM, aztreonam; CZA, ceftazidime/avibactam; AVI, avibactam; ME, major error; VME, very major error.

**Fig 3:**
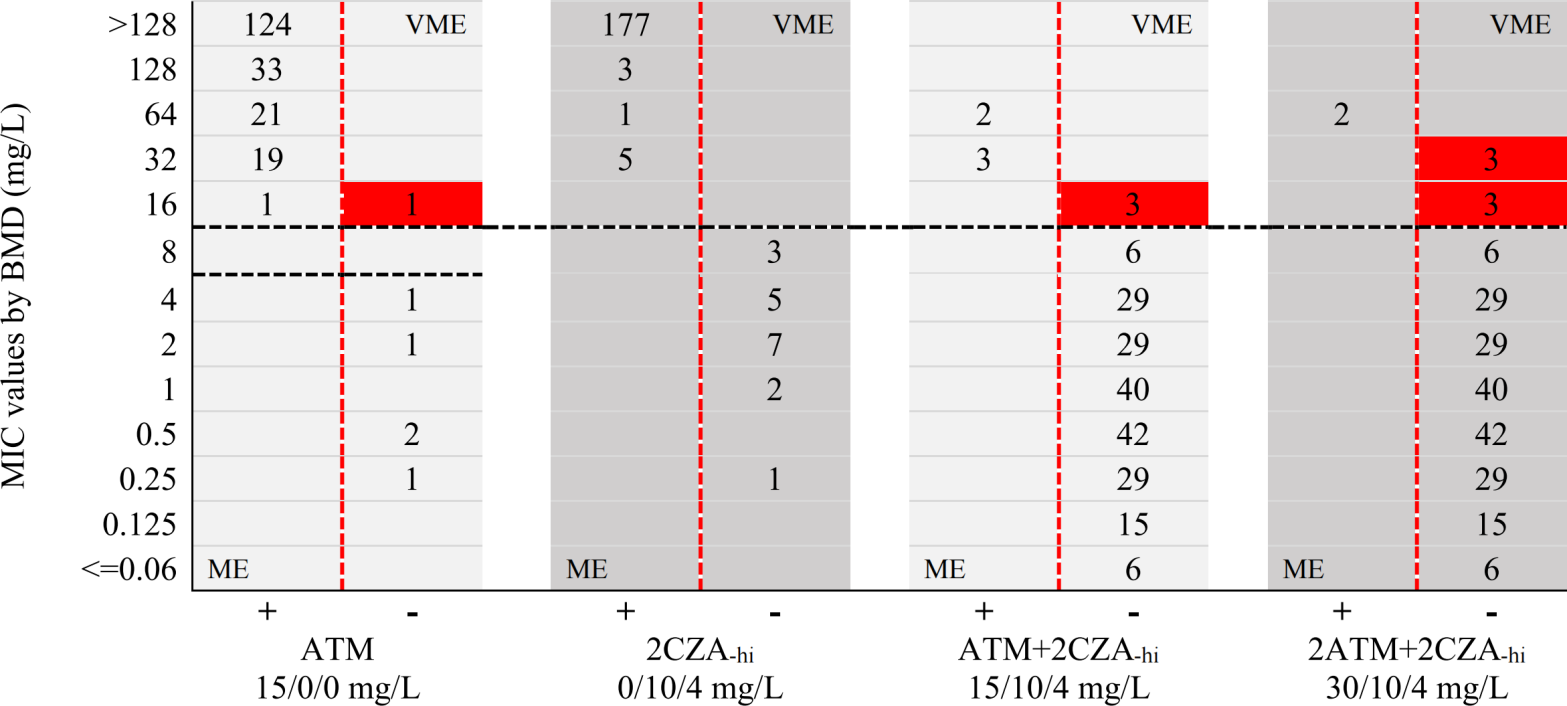
Comparsion scattergram between MIC values by BMD and phenotypic outcome by BDE-2mL against 204 *Enterobacterales* isolates. Black dash lines indicate the CLSI breakpoints for ATM and CZA, and the PK/PD breakpoints for ATM/AVI. Red dash lines are the boundary of growth (+) and no-growth (–) phenotypic outcomes by BDE. Red backgrounds indicate the VMEs observed in BDE. ATM, aztreonam; CZA, ceftazidime/avibactam; AVI, avibactam; ME, major error; VME, very major error.

For BMD, 96.1% (196/204) and 3.9% (8/204) of *Enterobacterales* were susceptible and resistant to aztreonam/avibactam, respectively. The susceptibility test of the BDE-5mL showed that the susceptibility rates and resistance rates to ATM+CZA_-lo_ tube (6/6/4 mg/L) were 95.6% (195/204) and 4.4% (9/204), whereas the rates to 2ATM+CZA_-lo_ tube (12/6/4 mg/L) were 96.1% (196/204) and 3.9% (8/204), respectively (Fig. 2). For the BDE-2mL, the susceptibility rates and resistance rates were 97.5% (199/204) and 2.5% (5/204) to ATM+2CZA_-hi_ tube (15/10/4 mg/L), whereas these were 99.0% (202/204) and 1.0% (2/204) to 2ATM+2CZA_-hi_ tube (30/10/4 mg/L), respectively (Fig. 3).

### Comparison of BDE-5mL and BMD

For the BDE-5mL test, the CA of the ATM tube was 100% (204/204) for all *Enterobacterales* (*n* = 204), with no ME and VME; the CA of the CZA_-lo_ tube was 99.5% (203/204), with 5.6% ME (1/18) and no VME; the CA of the ATM+CZA_-lo_ tube (6/6/4 mg/L) was 99.5% (203/204), with 0.5% ME (1/196) and no VME; the CA of the 2ATM+CZA_-lo_ tube (12/6/4 mg/L) was 100% (204/204), with no ME and VME. For 30 of the KPC+MBL-positive isolates, the CA values for ATM, CZA_-lo_, ATM+CZA_-lo_, and 2ATM+CZA_-lo_ tubes were all 100% (30/30), with no ME and VME. For 35 of the KPC-positive isolates, the CA values for ATM, ATM+CZA_-lo_, and 2ATM+CZA_-lo_ tubes were all 100% (35/35), with no ME and VME; the CA of the CZA_-lo_ tube was 97.1% (34/35), and the ME and VME were 6.7% (1/15) and 0%, respectively. For 139 of the MBL-positive isolates, the CA values for ATM, CZA_-lo_, and 2ATM+CZA_-lo_ tubes were all 100% (139/139), with no ME and VME; the CA of the ATM+CZA_-lo_ tube was 99.3% (138/139), and the ME and VME were 0.7% (1/137) and 0%, respectively (Table 2). The MIC of ceftazidime/avibactam for a *bla*_KPC-2_-positive *K. pneumoniae* isolate was 8 mg/L (susceptible) by BMD, but by BDE, the CZA_-lo_ tube was resistant. The MIC of aztreonam/avibactam for an *Enterobacter cloacae* isolate harboring *bla*_NDM-1_ was 4 mg/L (susceptible) by BMD, but by BDE, the ATM+CZA_-lo_ tube was resistant (Fig. 2; Table 4).

**TABLE 2:** Phenotypic outcomes of 204 *Enterobacterales* by broth disk elution of 5-mL CAMHB^*a*^

Strains	Antimicrobial agents	No. of isolates		% of isolates (No. of isolates/total no. of isolates)		
		S	R	CA	ME	VME
All *Enterobacterales* (*n* = 204)	ATM (6/0/0 mg/L)	5	199	100 (204/204)	0	0
	CZA_-lo_ (0/6/4 mg/L)	18	186	99.5 (203/204)	5.6 (1/18)	0
	ATM+CZA_-lo_ (6/6/4 mg/L)	196	8	99.5 (203/204)	0.5 (1/196)	0
	2ATM+CZA_-lo_ (12/6/4 mg/L)	196	8	100 (204/204)	0	0
KPC positive (*n* = 35)	ATM (6/0/0 mg/L)	0	35	100 (35/35)	0	0
	CZA_-lo_ (0/6/4 mg/L)	15	20	97.1 (34/35)	6.7 (1/15)	0
	ATM+CZA_-lo_ (6/6/4 mg/L)	29	6	100 (35/35)	0	0
	2ATM+CZA_-lo_ (12/6/4 mg/L)	29	6	100 (35/35)	0	0
MBL positive (*n* = 139)	ATM (6/0/0 mg/L)	5	134	100 (139/139)	0	0
	CZA_-lo_ (0/6/4 mg/L)	2	137	100 (139/139)	0	0
	ATM+CZA_-lo_ (6/6/4 mg/L)	137	2	99.3 (138/139)	0.7 (1/137)	0
	2ATM+CZA_-lo_ (12/6/4 mg/L)	137	2	100 (139/139)	0	0
KPC + MBL positive (*n* = 30)	ATM (6/0/0 mg/L)	0	30	100 (30/30)	0	0
	CZA_-lo_ (0/6/4 mg/L)	1	29	100 (30/30)	0	0
	ATM+CZA_-lo_ (6/6/4 mg/L)	30	0	100 (30/30)	0	0
	2ATM+CZA_-lo_ (12/6/4 mg/L)	30	0	100 (30/30)	0	0

^*a*^ ATM, aztreonam; CZA, ceftazidime/avibactam; KPC, *Klebsiella pneumoniae* carbapenemase; MBL, metallo-β-lactamase; S, susceptible; R, resistant; CA, categorical agreement; ME, major error; VME, very major error.

### Comparison of BDE-2mL and BMD

For the BDE-2mL test, the CA of the ATM tube for all 204 of *Enterobacterales* was 99.5% (203/204), and the ME and VME were 0% and 0.5% (1/199), respectively. The CA of the 2CZA_-hi_ tube was 100% (204/204), with no ME and VME; the CA of the ATM+2CZA_-hi_ tube (15/10/4 mg/L) was 98.5% (201/204), with 0% ME and 37.5% (3/8) VME; the CA of the 2ATM+2CZA_-hi_ tube (30/10/4 mg/L) was 97.1% (198/204), with 0% ME and 75% (6/8) VME. For 30 of the KPC+MBL-positive isolates, the CA values for ATM, 2CZA_-hi_, ATM+2CZA_-hi_, and 2ZTM+2CZA_-hi_ tubes were all 100% (30/30), with no ME and VME. For 35 of the KPC-positive isolates, the CA values for the ATM and 2CZA_-hi_ tubes were 100% (35/35), with no ME and VME; the CA of the ATM+2CZA_-hi_ tube was 91.4% (32/35), with 0% ME and 50% (3/6) VME; the CA of the 2ATM+2CZA_-hi_ tube was 89.6% (31/35), with 0% ME and 66.7% (4/6) VME. For 139 of the MBL-positive isolates, the CA of the ATM tube was 99.3% (138/139), with 0% ME and 0.7% (1/134) VME; the CA values for 2CZA_-hi_ and ATM+2CZA_-hi_ tubes were all 100% (139/139), with no ME and VME; the CA, ME, and VME for 2ATM+2CZA_-hi_ tubes were 98.6% (137/139), 0%, and 100% (2/2), respectively (Table 3). The MIC of aztreonam for a *bla*_NDM-5_-positive *K. pneumoniae* isolate was 16 mg/L (resistant) by BMD, but the BDE result of the ATM tube showed no bacterial growth (susceptible). The MICs of aztreonam/avibactam for three *bla*_KPC-2_-positive *K. pneumoniae* isolates were all 16 mg/L (resistant) by BMD, but the BDE results of the ATM+2CZA_-hi_ tubes showed no bacterial growth (susceptible). The MICs of aztreonam/avibactam for four *bla*_KPC-2_-positive *K. pneumoniae* isolates and two *bla*_NDM-5_-positive *E. coli* isolates were 16–32 mg/L (resistant) by BMD, but the BDE results of the 2ATM+2CZA_-hi_ tubes showed no bacterial growth (susceptible) (Fig. 3; Table 4).

**TABLE 3:** Phenotypic outcomes of 204 *Enterobacterales* by broth disk elution of 2-mL CAMHB^*a*^

Strains	Antimicrobial agents	No. of isolates		% of isolates (No. of isolates/total no. of isolates)		
		S	R	CA	ME	VME
All *Enterobacterales* (*n* = 204)	ATM (15/0/0 mg/L)	5	199	99.5 (203/204)	0	0.5 (1/199)
	2CZA_-hi_ (0/10/4 mg/L)	18	186	100 (204/204)	0	0
	ATM+2CZA_-hi_ (15/10/4 mg/L)	196	8	98.5 (201/204)	0	37.5 (3/8)
	2ATM+2CZA_-hi_ (30/10/4 mg/L)	196	8	97.1 (198/204)	0	75 (6/8)
KPC positive (*n* = 35)	ATM (15/0/0 mg/L)	0	35	100 (35/35)	0	0
	2CZA_-hi_ (0/10/4 mg/L)	15	20	100 (35/35)	0	0
	ATM+2CZA_-hi_ (15/10/4 mg/L)	29	6	91.4 (32/35)	0	50 (3/6)
	2ATM+2CZA_-hi_ (30/10/4 mg/L)	29	6	89.6 (31/35)	0	66.7 (4/6)
MBL positive (*n* = 139)	ATM (15/0/0 mg/L)	5	134	99.3 (138/139)	0	0.7 (1/134)
	2CZA_-hi_ (0/10/4 mg/L)	2	137	100 (139/139)	0	0
	ATM+2CZA_-hi_ (15/10/4 mg/L)	137	2	100 (139/139)	0	0
	2ATM+2CZA_-hi_ (30/10/4 mg/L)	137	2	98.6 (137/139)	0	100 (2/2)
KPC + MBL positive (*n* = 30)	ATM (15/0/0 mg/L)	0	30	100 (30/30)	0	0
	2CZA_-hi_ (0/10/4 mg/L)	1	29	100 (30/30)	0	0
	ATM+2CZA_-hi_ (15/10/4 mg/L)	30	0	100 (30/30)	0	0
	2ATM+2CZA_-hi_ (30/10/4 mg/L)	30	0	100 (30/30)	0	0

^*a*^ ATM, aztreonam; CZA, ceftazidime/avibactam; KPC, *Klebsiella pneumoniae* carbapenemase; MBL, metallo-β-lactamase; S, susceptible; R, resistant; CA, categorical agreement; ME, major error; VME, very major error.

**TABLE 4:** Summary of categorical errors for broth disk elution after repeat testing

Species	Carbapenemase	BMD MIC results (mg/L)			BDE-5mL results (mg/L)				BDE-2mL results (mg/L)			
		ATM	CZA	AZA	ATM (6/0/0)	CZA_-lo_ (0/6/4)	ATM+CZA_-lo_ (6/6/4)	2ATM+CZA_-lo_ (12/6/4)	ATM (15/0/0)	2CZA_-hi_ (0/10/4)	ATM+2CZA_-hi_ (15/10/4)	2ATM+2CZA_-hi_ (30/10/4)
*Klebsiella pneumoniae*	KPC-2	>128	8	4	+	**+^*b*^**	–	–	+	–	–	–
*Enterobacter cloacae*	NDM-1	>128	>128	4	+	+	**+^*b*^**	–	+	+	–	–
*Klebsiella pneumoniae*	NDM-5	16	>128	0.5	+	+	–	–	–^*c*^	+	–	–
*Escherichia coli*	NDM-5	>128	>128	32	+	+	+	+	+	+	+	–
*Klebsiella pneumoniae*	KPC-2	>128	32	32	+	+	+	+	+	+	+	–
*Escherichia coli*	NDM-5	32	>128	32	+	+	+	+	+	+	+	–
*Klebsiella pneumoniae*	KPC-2	>128	32	16	+	+	+	+	+	+	–	–
*Klebsiella pneumoniae*	KPC-2	>128	64	16	+	+	+	+	+	+	–	–
*Klebsiella pneumoniae*	KPC-2	>128	32	16	+	+	+	+	+	+	–	–

^*a*^ +, Growth; –, no growth; ATM, aztreonam; CZA, ceftazidime/avibactam; AZA, aztreonam/avibactam.

^*b*^ Bold indicates the MEs observed in BDE.

^*c*^ Grey backgrounds indicate the VMEs observed in BDE.

## DISCUSSION

Infections caused by MBL-producing *Enterobacterales* are currently a major public health threat worldwide (26). As a novel drug combination, aztreonam/avibactam has been shown to have strong antibacterial activity against MBL-producing *Enterobacterales* and can be used for the treatment of infections caused by these pathogens (27–29); however, Livermore et al. had reported 18 resistant isolates to aztreonam/avibactam in MBL-producing *Enterobacterales*, similar to reports by Bhatnagar et al. and Ma et al., in which the MICs of all the resistant isolates were 16–32 mg/L (27, 30, 31). So, it is crucial for clinical microbiology laboratories to provide aztreonam/avibactam results to clinicians to avoid treatment failures due to the infections caused by MBL-producing *Enterobacterales*. Although aztreonam/avibactam is currently in phase III clinical trials, the combination of aztreonam with ceftazidime/avibactam can be achieved. As for the susceptibility testing, there have been several studies on aztreonam in combination with ceftazidime/avibactam, including the use of modified Etest/disk diffusion method to detect the synergistic effect of aztreonam and ceftazidime/avibactam, which required that the distance between the ceftazidime/avibactam Etest strip and the center of the aztreonam disk should be 15 mm, and that the aztreonam disk should be placed near the breakpoint MIC of the Etest strip; and the CA of modified Etest/disk diffusion method was only about 80% (32, 33). In addition, some scholars also used the gradient strip stacking method and the gradient strip crossing method to determine the synergistic effect of aztreonam and ceftazidime/avibactam, and the CA of the gradient strip-based synergy testing was 82% to 94%, with 0%–5% ME and no VME (19). However, all of the above methods had drawbacks such as poor repeatability of results from different operators or high prices (19, 32–34). Recent studies detected the combined effect of aztreonam with ceftazidime/avibactam using the disk stacking plus micro-elution method, which could obtain results after 8 hours, but with only 92.8% sensitivity and 93.8% categorical agreement (35); however, our study showed that the CA of BDE-5mL was 99.5% to 100%, with 0%–0.5% ME and no VME. In this study, the combination of ceftazidime/avibactam at different concentrations with aztreonam was used to evaluate its methodological detection performance, with the aim of finding a susceptibility testing method with a simple operation, a low cost, and easy-to-determine results for clinical microbiology laboratories. As CLSI has not established the breakpoint standard for aztreonam/avibactam, the susceptible breakpoint selected for aztreonam/avibactam was ≤8 mg/L, based on the PK/PD studies of Cornely et al. and Singh et al, and the reliability of this breakpoint was also confirmed by the research of Sader et al. (10, 22, 23). In addition, unlike the studies of Harris et al. and Khan et al., who evaluated the performance of BDE using only one concentration of ATM-CZA tube (18, 19), the concentration of aztreonam/avibactam in our study covers the MIC ranges of PK/PD breakpoints, including 6/4–12/4 mg/L and 15/4–30/4 mg/L, which matched the constant avibactam concentration of 4 mg/L used in BMD to better evaluate the detection performance of aztreonam/avibactam in different concentration ranges.

Compared with the BMD, in the tests of BDE-5mL, the results of ATM+CZA_-lo_ tube of 6/6/4 mg/L and 2ATM+CZA_-lo_ tube of 12/6/4 mg/L all achieved the acceptable standards of CA, ME, and VME, as well as the CA, VME, and ME for different types of carbapenemase-producing *Enterobacterales*. Thus, it was suggested that the BDE-5mL method was suitable for detecting the susceptibility of aztreonam/avibactam for different carbapenemase-producing *Enterobacterales* strains including MBL producers and non-MBL producers, especially the 2ATM+CZA_-lo_ tube of 12/6/4 mg/L, in which the CA achieved 100% for all types of carbapenemase-producing strains and was superior to the ATM+CZA_-lo_ tube of 6/6/4 mg/L. The previous study by Harris et al. mainly evaluated the performance of the BDE method for MBL-producing strains, and the CA, ME, and VME values were 98.1%, 2%, and 0%, respectively, which are consistent with the results of this study (18). However, our study also evaluated the performance of the BDE method in detecting the susceptibility of KPC-producing and KPC+MBL-producing strains to aztreonam/avibactam, which broadened the application objects of the BDE method. As for the tests of BDE-2mL, the CA values for the ATM+2CZA_-hi_ tube (15/10/4 mg/L) and 2ATM+2CZA_-hi_ tube (30/10/4 mg/L) were all >95%, but the VMEs were 37.5% and 75%, respectively, far beyond the acceptable range. Further analysis showed that the VMEs of the ATM+2CZA_-hi_ tubes (15/10/4 mg/L) were mainly caused by three *K. pneumoniae* strains carrying *bla*_KPC-2_, and the VMEs of the 2ATM+2CZA_-hi_ tubes (30/10/4 mg/L) were mainly concentrated in four *K. pneumoniae* carrying *bla*_KPC-2_ and two *E. coli* carrying *bla*_NDM-5_, indicating that the ATM+2CZA_-hi_ tube (15/10/4 mg/L) and 2ATM+2CZA_-hi_ tube (30/10/4 mg/L) may overestimate the susceptibility of aztreonam/avibactam for detecting antimicrobial activity against the KPC-producing *Enterobacterales* isolates. Significantly, the presented data with BDE-2mL suggested higher VMEs when the isolate MIC approaches the BDE concentration tested; however, the BDE-5mL (12/6/4 mg/L) showed no VME. Further analysis suggested that this may be because the BDE-2mL concentration (15/10/4 mg/L) was closer to the isolates with a MIC of 16 mg/L than the BDE-5mL (12/6/4 mg/L) , so that it was easier to be misclassified especially for the isolates with a MIC of 16 mg/L. On the other hand, aztreonam/avibactam-resistant isolates with MICs ranging from 16 to 32 mg/L were only six in this study; therefore, when more resistant isolates with MICs of 16–32 mg/L can be collected, further evaluation of the two concentrations (12/6/4 mg/L and 15/10/4 mg/L) is needed to reduce bias. In this study, we tested seven KPC-variant-producing isolates by using the BDE method (four KPC-33 producers, two KPC-71 producers, and one KPC-135 producer), and the CA rate, compared to BMD, reached 100%, indicating that the BDE method also had superior performance in detecting the susceptibility of KPC-variant producers to aztreonam/avibactam.

Moreover, there are some limitations in this study. First, there were only eight *Enterobacterales* clinical isolates that were resistant to aztreonam/avibactam, which may introduce some bias in the interpretation of the results. Second, the disks used in this study only came from one manufacturer, because it seems that only Mast Group Ltd. (UK) has the 10/4-µg ceftazidime/avibactam disks. To ensure comparability of results, we conducted tests using the disks of 10/4-µg ceftazidime/avibactam and 30/20-µg ceftazidime/avibactam from the same manufacturer. Third, we only evaluated the performance of BDE in *Enterobacterales*.

In conclusion, according to the results of this study, we can find that the BDE-5mL combined with aztreonam disk and ceftazidime/avibactam disk is a simple, cost-effective, and reliable method that can be used to determine the susceptibility of *Enterobacterales* to aztreonam/avibactam, especially the 2ATM+CZA_-lo_ tubes with a final concentration of 12/6/4 mg/L of aztreonam/ceftazidime/avibactam. Thus, it is expected to be promoted and used in clinical microbiology laboratories.
